# Health – related quality of life of Kuwaiti women with breast cancer: a comparative study using the EORTC Quality of Life Questionnaire

**DOI:** 10.1186/1471-2407-9-222

**Published:** 2009-07-08

**Authors:** Shafika A Alawadi, Jude U Ohaeri

**Affiliations:** 1 Department of Medicine, Faculty of Medicine, Kuwait University, Kuwait; 2 Department of Psychiatry, Psychological Medicine Hospital, Gamal Abdul Naser Road, PO Box 4081, Safat, 13041, Kuwait

## Abstract

**Background:**

The Kuwaiti perspective on quality of life (QOL) in breast cancer is important because it adds the contribution from a country where the disease affects women at a relatively younger age and seems to be more aggressive. We used the EORTC QLQ – C30 and its breast-specific module (BR-23) to highlight the health-related QOL of Kuwaiti women with breast cancer, in comparison with the international data, and assessed the socio-demographic and clinical variables that predict the five functional scales and global QOL (GQOL) scale of the QLQ – C30.

**Methods:**

Participants were consecutive clinic attendees for chemotherapy, in stable condition, at the Kuwait Cancer Control Center.

**Results:**

The 348 participants were aged 20–81 years (mean 48.3, SD 10.3); 58.7% had stages III and IV disease. Although the mean scores for QLQ – C30 (GQOL, 45.3; and five functional scales, 52.6%–61.2%) indicated that the patients had poor to average functioning, only 5.8% to 11.2% had scores that met the </= 33% criterion for problematic functioning, while 12.0% to 40.0% met the >66% criterion for more severe symptoms. Most (47.8%–70.1%) met the >66% criterion for "good functioning" on the BR-23 functional scales. The mean scores of the QLQ – C30 indicated that, despite institutional supports, Kuwaiti women had clinically significantly poorer global QOL and functional scale scores, and more intense symptom experience, in comparison with the international data (i.e., </= 10% difference between groups). For the BR-23, Kuwaiti women seemed to have clinically significantly better functional scale scores, but more severe symptoms, especially systemic side effects and breast symptoms. Younger women had poorer HRQOL scores. In regression analysis, social functioning accounted for the highest proportion of variance for GQOL.

**Conclusion:**

The relatively high number that met the criterion for good functioning on the functional scales is an evidence base to boost national health education about psychosocial prognosis in cancer. In view of the poor performance on the symptom scales, clinicians treating Kuwaiti women with breast cancer should prepare them for the acute toxicities of treatment and address fatigue. The findings call for the institution of a psycho-oncology service to address psycho-social issues.

## Background

In the field of medicine, one popular definition of the construct of quality of life (QOL), based on the World Health Organization's definition of health, is that it concerns physical, psychological and social functioning, incorporating positive aspects of wellbeing as well as negative aspects of disease [1] Health – related QOL (HRQOL) is that aspect attributable to the disease and its treatment, and generally consists of assessments of functional health status (i.e., limitations on physical, psychological and social functioning imposed by the disease) and global wellbeing [2].

Although there has been considerable research on the QOL of women with breast cancer [3], the results may not be significantly affecting clinical decision – making because the clinical significance of findings is not specified [4,5]. More studies are therefore needed to make QOL assessment feasible, understandable and scientifically viable in oncology research and practice [5]. In particular, HRQOL assessments in oncology facilitate doctor – patient communication, they point to areas where patients may experience serious problems, they can be used as diagnostic tools for problem- oriented follow – up care, and the data are strong predictors of survival [6,7].

These issues are highly relevant in Arab countries, such as Kuwait, where there is paucity of studies on QOL in cancer [8,9], and the development of modern oncology services has led to improved survival rates, thereby making the disease to be a chronic condition. In addition, there are indications that different cultural groups may emphasize different aspects of their QOL [10]. For example, analysis of a large international database of the European Organization for Research and Treatment in Cancer Quality of Life Questionnaire (EORTC QLQ – C30) indicated that, compared with subjects from the UK, physical and social functioning were less important in predicting the global QOL of subjects from Islamic countries, while cognitive functioning was more influential for South Asia and Latin America [10]. This obviates the need to assess the local perspectives on international QOL instruments, so that the data can be used to guide the choice of interventions and for cross-cultural comparisons to contribute to the emerging QOL theory [11]. Accordingly, researchers have sought to highlight the factors that predict HRQOL among women with breast cancer. The common findings are as follows: First, younger age and chemotherapy are risk factors for diminished QOL [12-16]. Second, the association of stage of breast cancer with QOL is more controversial, with some authors finding no significant relationship [15,17], while others report significant impact, in line with expectation of known groups validity [3,9,18,19]. Also controversial is the impact on QOL, of the type of breast surgery [12,14,15], and time since surgery and chemotherapy [12,20]. Third, disease – free women tend to have global QOL scores similar to the general population [21].

In this study, we sought to present an Arab perspective on the above issues, by using the EORTC QLQ – C30 [17] and its breast cancer specific module (BR -23) [18] to assess women attending the outpatient service of the Kuwait Cancer Control Center (KCCC) for chemotherapy. We chose the EORTC QLQ for the following reasons. First, although it was articulated in Europe, it has been found to be reliable and valid in diverse cultures, including, the United Arab Emirates [9], Iran [19], Turkey [22], Japan [23], India [13], China [24], Korea [25], and Nigeria [26]. Hence, there is an impressive body of international data with which to compare our results. Second, an Arabic translation of the questionnaire already exists, approved by the authors of the instrument.

The Kuwaiti perspective is important because it adds the contribution from a country where the pathobiological features of the disease indicate that it affects women at a relatively younger age and it seems to be more aggressive than what is currently seen in Europe, North America, Australia, and parts of Asia [27]. In addition, the highly developed cancer service at the KCCC is easily accessible to all in this materially affluent city – state, and is provided free – of – charge to all Kuwaiti nationals. Since met needs for care have been known to be associated with QOL [28,29], one wonders whether these favorable social/institutional care factors could contribute to make the HRQOL of Kuwait women to be comparable to the international data, despite the unfavorable factors (e.g., pathobiological features mentioned above, and use of chemotherapy). In Kuwait, breast cancer is the most common malignancy among women, accounting for 30.3% of all cancer types, and death occurs in approximately 43% of patients [27].

### Objectives

The objectives of the study were as follows: First, to highlight the HRQOL scale scores for Kuwaiti women in stable clinical condition with breast cancer, in comparison with the international data. Second, to examine the association of HRQOL with socio-demographic characteristics, stage of disease, type of treatment received in the past (i.e., surgery and radiotherapy), and duration since last treatment (for chemotherapy, surgery and radiotherapy). Third, to assess the variables that predict the global health status/QOL scale (GQOL) and the five functional scales of the QLQ – C30 (i.e., physical, role, emotional, cognitive, and social functioning), in comparison with the international data. In particular, we examined the relationship between GQOL, the five functional scales and the symptom scales/items [10].

Based on the literature, we hypothesized that despite unfavorable biological/treatment factors (i.e., their relatively younger age, more aggressive tumor and the fact that most were on chemotherapy at the time of assessment) [12-16], Kuwaiti women would have comparable HRQOL scores with the international data because of favorable social/care factors [28,29]. Second, there is significant association between HRQOL scale scores and other demographic variables, stage of disease, time since last treatment, and previous surgery and radiotherapy. Third, as indicated by data from other Islamic countries [10], physical and social functioning would be less important in predicting the GQOL. On the other hand, since the occurrence of physical symptoms can cause a change in QOL [30] the symptom scales will be prominent predictors of GQOL.

## Methods

### Subjects and setting of the study

The subjects were consecutive attendees at the outpatient clinic of the medical oncology department of the KCCC who fulfilled the study's inclusion criteria. They were attending follow-up clinic appointment for chemotherapy. No restrictions were made with regard to age and type of chemotherapy [18]. In order to make our findings comparable with those of others, participants were in stable clinical condition (i.e., were not in acute pain, could move about unassisted, did not require hospitalization), had clear consciousness, could easily follow the interview process, and could independently give consent to participate in the study [31]. In this culture, female patients are, as a rule, accompanied to hospital by family members who live with them [32]. Consent was also obtained from the family members; but the patients were interviewed privately in one of the clinic rooms designated for the study. Patients involved in this study were Kuwaiti nationals.

The KCCC is the national center for treatment of cancer, and houses the National Cancer Registry, the Kuwait National Cancer Control Program (in collaboration with the WHO), the Radiation Oncology and Nuclear Medicine Departments, as well as the Medical, Pediatric, and Surgical Oncology Departments, and diagnostic facilities. All cancer cases in the country are referred there. It is an ultra-modern facility, with adequate facilities for diagnosis and treatment of cancer. It has a large complement of specialists in the various fields of oncology. There are daily outpatient clinics and 190 inpatient beds. All services are rendered free – of – charge to Kuwaitis, while others pay nominal fees.

Since this study was not a drug trial, we included patients with all types of treatment, as determined by their doctors. Hence, patients received a wide variety of chemotherapy.

### The EORTC QLQ – C30 and BR – 23

The EORTC QLQ – C30 is a 30 – item generic HRQOL instrument designed to assess cancer patients' physical, psychological and social functioning [17,31]. It is composed of nine multi-item scales: 5 functional scales (listed above), a global QOL scale (GQOL), and three symptom scales (fatigue, pain, nausea & vomiting). In addition, there are five single item symptom scales (dyspnea, sleep disturbance, appetite loss, constipation, & diarrhea); and a final item evaluates the perceived financial impact of the disease. In the version used for this study (version 3), the first 28 items are rated on a response scale of "not at all (1), to "very much" (4). The time frame is the present moment. For items 29 (on overall general health) and 30 (on overall QOL), the response options range from "very poor" (1) to "excellent" (7), and the time frame is "during the past week".

The 23 – item breast cancer -specific module, the QLQ -BR -23 [18], consists of two multi-item functional scales (body image and sexual functioning), three symptom scales (systemic side effects, breast symptoms, & arm symptoms), and three single item scales on sexual enjoyment, future perspectives, and upset by hair loss. The response options are "not at all" (1), to "very much" (4), and the time frame is "during the past week", except for the sexual items ("during the past four weeks"). The scoring algorithm recommended by the EORTC [33], was used to transform the responses to values on a scale of 0 – 100%. For the functional scales and GQOL, a higher score corresponds to better functioning and QOL. For symptom scales, a higher score corresponds to more frequent and/or more intense symptoms. A problematic group is defined as one with a GQOL or functional scale score of 33 or less, and a symptom scale score of 66 or more on the QLQ – C30 and QLQ – BR-23 [20,28].

We assessed the internal consistency of the questionnaires by estimating the Cronbach's alpha values of the multi-item scales, based on the recommendation of > 0.70. The alpha coefficients were highly significant: for the first 28 items of the QLQ – C30, it was 0.94 (split half reliability = 0.84). For the 23 items of the BR-23, the alpha coefficient was 0.92 (split -half reliability = 0.81).

### Data collection procedure

At the preliminary stage of the study, the EORTC Quality of Life Unit in Belgium, kindly sent us the questionnaires (English & Arabic translations) and the scoring manual.

The study was carried out in compliance with the Helsinki Declaration. Hence, ethical approval for the work was obtained from the institutional review panel of the KCCC. In addition, patients and their family caregivers gave verbal informed consent to participate in the study. They were duly informed that there would be no negative consequences for declining to participate. As is well known in our culture for such non-invasive studies [32], all families approached freely consented to participate in the study, especially as the approach was made by clinic staff in charge of the cases.

All assessments were based on private interviews by a trained female Arab research assistant, who has had previous experience in administering QOL questionnaires. The research assistant read out the questions to the participants and rated their responses. The criteria for staging disease by the doctors were those of the American Joint Committee on Cancer [34].

### Data analysis

Data were analyzed by the Statistical Package for Social Sciences, version 15 for windows (SPSS Inc., Chicago, Illinois). The scale scores of the QLQ – C30 and BR-23 were computed as recommended [33]. In order to test the first hypothesis, we used scale scores adjusted for age, occupation, marital status and stage of cancer, to compare our data with similar reports from the neighboring United Arab Emirates (UAE), various European countries, and Asia. These reports were located by a limited Medline search for recent English language papers that concerned patients in stable condition from various regions of the world, using the key words: EORTC QLQ – C30; breast cancer. Comparisons were done by effect size calculations and by adopting the operational definition of a clinically meaningful (significant) difference of 10% between groups [5,35]. Where effect size calculations were done for HRQOL scales, we defined a clinically significant difference as ≥ 0.5 [36]. For the functional scales and the GQOL, we defined subjects with problematic functioning as those who scored ≤ 33%, while subjects in good condition scored >66%. For symptom scales, subjects scoring ≤ 33% were judged as having less severe symptoms, while those scoring > 66% had more intense symptoms [20]. We have used the ≤ 33% cut-off scale score to categorize the patients because that score was suggested from an empirical general population study [37], rather than theoretical speculation. For the second hypothesis, we analyzed the association of scale scores with socio-demographic and clinical variables, by Pearson's correlations, t-tests and one-way ANOVA. The strength of these relationships was tested in multivariate analysis by analysis of covariance (ANCOVA). For the third hypothesis, we used multiple (stepwise) regression analyses to assess the predictors of the functional scales and GQOL scale of the QLQ – C30. With each of the functional scales as dependent variable, the independent (predictor) variables were entered in blocks, starting from the background socio-demographic and clinical variables, followed by the other functional scales, then the symptom scales, and finally, the single item scales of the QLQ – C30. In the case of the GQOL, the scales of the BR-23 were also entered as independent variables. Missing data were handled by deleting cases, analysis- by- analysis. Where there was multiple testing, we used a Bonferroni correction of P < 0.01. Otherwise, the level of significance was set at P < 0.05, and all tests were two-tailed.

## Results

### Socio-demographic and clinical characteristics (Table 1)

**Table 1:** Socio – demographic and clinical characteristics of breast cancer patients

Variables	No. of subjects (%)
Age groups (yrs): N = 345	
20 – 39	60 (17.4)
40 – 59	232 (67.2)
60 – 81	53 (15.4)
Mean (SD): 48.3 (10.3); Median, & Mode = 46.0	
Marital status: N = 347	
Married	312 (89.9)
Single	11 (3.2)
Divorced/widow	24 (6.9)
Occupational status: N = 343	
Housewife/retired	75 (21.9)
Low skill work (e.g., clerk)	45 (13.1)
Medium skill (e.g., school teacher)	191 (55.7)
High skill work (e.g., doctor, lawyer, engineer)	32 (9.3)
Stage of cancer (N = 344)	
I	24 (7.0)
II	118 (34.3)
III	124 (36.0)
IV	78 (22.7)
Type of treatment received	
Chemotherapy	342 (98.3)
Radiotherapy	73 (21.0)
Surgery	204 (58.6)
Duration since last chemotherapy (N = 341)	
Past month	315(92.4)
1 month to 3 months	13 (3.8)
> 3 months	13 (3.8)
Duration since last radiotherapy: N = 69	
Past month	48(70.7)
1 – 6 months	4 (5.7)
> 6 months	17 (24.6)
Duration since last surgery: N = 202	
Past month	49(24.3)
1 – 3 months	78(38.6)
> 3 months	75 (37.1)

Those with stage IV disease were older (51.0) than those with other stages. This reached significance in comparison with stage III patients (46.9), (F = 2.7, df = 3/341, P < 0.05).

Over a period of one year (2007/2008), 348 women fulfilled the study's inclusion criteria and freely agreed to participate. They were aged 20 – 81 years (mean, 48.3, SD 10.3; median & mode = 46). Only six subjects (1.7% of 345) were aged < 30 years, while 82 (23.8%) were aged > 55 years. This age pattern is much similar to breast cancer clinic samples in previous reports from Kuwait [27,38]. The participants were predominantly married (89.9%), and in full employment (78.1%). Majority (58.7) were being treated for advanced disease (i.e., stages III & IV). Apart from chemotherapy, majority (58.6%) had had surgery, while 21% had received radiotherapy. Most (92.4%) had received a course of chemotherapy in the past month.

### Profile of HRQOL scale scores (Tables 2 and 3)

**Table 2:** QLQ – C30 & BR -23 unadjusted scale scores, percentage of subjects with problems & in good condition^a^.

Variables	N	No. of items	Mean (SD)	95% C.I.	% scoring < 33.3%^c^	% scoring ≥ 66.7%^c^
C-30 Functional scales^b^						
Global QOL/general health	340	2	45.3(15.3)	43.7 – 46.9	6.2	10.9
Physical functioning	330	5	52.6(18.8)	50.6 – 54.6	11.2	25.2
Role functioning	340	2	55.1(21.4)	52.7 – 57.5	7.6	39.1
Emotional	323	2	60.3(22.5)	57.7 – 62.9	8.4	45.2
Cognitive	342	4	59.9(23.9)	57.3 – 62.5	5.8	51.2
Social	339	2	61.2(22.7)	58.8 – 63.6	6.5	52.8
C – 30 symptoms/scales^c^						
Fatigue	331	3	38.9(21.3)	36.5 – 41.3	29.0	13.6
Nausea & vomiting	334	2	30.2(24.5)	27.6 – 32.8	48.5	12.0
Pain	334	2	43.8(21.7)	41.4 – 46.2	20.1	27.5
Dyspnoea	335	1	42.4(27.8)	39.4 – 45.4	18.8	40.0
Sleep disturbance	338	1	42.7(28.2)	39.7 – 45.7	18.3	39.3
Appetite loss	339	1	37.5(28.0)	34.5 – 40.5	25.1	32.7
Constipation	345	1	27.8(28.2)	24.8 – 30.8	42.0	22.0
Diarrhea	343	1	22.1(27.5)	19.1 – 25.1	54.8	19.2
Financial impact	344	1	31.2(25.2)	28.4 – 34.0	29.1	19.8
QOL – BR23: Functional scales^b^						
Body image	299	4	61.8(23.3)	59.0 – 64.6	7.0	47.8
Sexual functioning	303	2	69.9(23.6)	67.1 – 72.7	1.3	68.0
Sexual enjoyment	221	1	61.5(23.0)	58.3 – 64.7	2.3	70.1
Future perspective	334	1	59.5(31.9)	56.1 – 62.9	11.7	64.7
BR23: Symptom scales^c^						
Systemic side effects	300	7	40.1(17.5)	38.1 – 42.1	29.3	6.7
Breast symptoms	293	4	35.6(25.4)	32.6 – 38.6	42.3	14.3
Arm symptoms	318	3	38.2(23.4)	35.6 – 40.8	33.0	13.8
Upset by hair loss	331	1	44.8(29.6)	41.6 – 48.0	17.2	40.8

^a^For functional scales, subjects scoring < 33.3% have problems; those scoring ≥ 66.7% have good functioning. For symptom scales/symptoms, subjects scoring < 33.3% have good functioning; those scoring = 66.7% have problems

^b ^For functional scales, higher scores indicate better functioning

^c ^For symptom scales, higher scores indicate worse functioning

**Table 3:** QLQ – C30 & BR – 23 adjusted scale scores*

Variables	N	Mean (SE)	95% C.I.
QLQ C-30 functional scales			
Global QOL/general health	331	45.0(0.8)	43.4 – 46.7
Physical functioning	316	52.7(1.1)	50.6 – 54.8
Role functioning	331	55.1(1.2)	52.7 – 57.4
Emotional	314	60.2(1.8)	57.7 – 62.7
Cognitive	333	59.4(1.3)	57.3 – 62.5
Social	330	61.3(1.3)	58.9 – 63.8
QLQ C – 30 symptoms scales			
Fatigue	322	38.9(1.2)	36.5 – 41.2
Nausea & vomiting	326	30.2(1.3)	27.5 – 32.8
Pain	325	43.8(1.2)	41.4 – 46.2
Dyspnoea	327	42.1(1.5)	39.1 – 45.1
Sleep disturbance	329	42.7(1.6)	39.5 – 45.6
Appetite loss	330	37.4(1.5)	34.4 – 40.4
Constipation	336	27.8(1.5)	24.8 – 30.8
Diarrhea	334	21.9(1.5)	19.0 – 24.9
Financial impact	335	31.2(1.4)	28.5 – 33.9
QOL – BR23: functional scales			
Body image	290	61.8(1.4)	59.2 – 64.5
Sexual functioning	295	70.2(1.3)	67.6 – 72.9
Sexual enjoyment	215	61.8(1.6)	58.7 – 64.9
Future perspective	325	59.7(1.8)	56.3 – 63.1
QLQ – BR23: Symptom scales			
Systemic side effects	292	40.2(1.0)	38.2 – 42.2
Breast symptoms	284	35.3(1.5)	32.4 – 38.3
Arm symptoms	309	38.2(1.3)	35.6 – 40.8
Upset by hair loss	323	44.4(1.6)	41.2 – 47.6

* Scores adjusted for age, stage of cancer, marital status, and occupation

For functional scales, higher scores indicate better functioning

For symptom scales, higher scores indicate worse functioning

Of the QLQ – C30 scales, the patients seemed to perform poorly to averagely on both the symptom scales and the functional health status scales. Hence, of the functional scales, the mean score for the global QOL scale (GQOL) was less than 50%, while the range of mean scores for the five scales was 52.6% to 61.2%, indicating predominantly low level of general wellbeing with average level of functional health status. Judging by the cut-off score of 66%, the best domains of functioning were "cognitive" and "social", where just over one-half of participants had good level of functioning. The domains with poorest functioning were general wellbeing and physical functioning, where only 10.9% – 25.2% had good level of functioning. It is noteworthy that only 6.5% to 11.2% had scores that met operational criterion for problematic functioning in the functional domains.

They performed much worse on the symptom scales, because 13.6% to 40.0% met the operational criterion for significantly high levels of symptoms. The commonest problem areas (i.e., over one- quarter had "problematic" scores) were, pain, dyspnoea, sleep disturbance and poor appetite. However, 18.3% to 54.8% met the operational criterion for "good" functioning for the symptom scales. The patients performed best on the functional scales of the breast specific scales (BR-23) because most (47.8 – 70.1%) met the operational criterion for "good" functioning. In particular, about two-thirds had good sexual functioning and were optimistic about their future health, while almost one-half expressed little or no problems with their body image.

Of the BR-23 symptom scales, the commonest area with significantly intense level of symptom experience was hair loss. However, 17.2% – 42.3% seemed to be relatively free from intense levels of symptoms for the breast -specific symptom scales.

### Factors associated with HRQOL scale scores

Using Pearson's correlation analysis, the correlation of age with scale scores was low (i.e., < 0.2). The correlations were significant and positive for the following functional scales: emotional functioning, cognitive functioning, social functioning, sexual enjoyment, body image (r ranged from 0.12 to 0.18, P < 0.02), and sexual functioning (r = 0.21, P < 0.0001). The correlations were significant and negative for the following symptom scales: systemic side effects, breast symptoms, arm symptoms, upset by hair loss (r ranged from -0.14 to -0.19, P < 0.01). In other words, for the functional and symptom scales, older patients tended to have better functioning and less intense symptoms than the younger patients.

Using Pearson's correlation analysis for the symptom scales of the QLQ – C30, the correlation with GQOL was highest for fatigue (r = 0.21, P < 0.0001). In one-way ANOVA, marital and occupational status were not significantly associated with scale scores (P > 0.05). Subjects with advanced disease tended to have worse functioning. This reached significance for the following: role functioning (stage IV < stage II, F = 3.8, df = 3/335, P < 0.01), diarrhea (stage IV < stages I & II, F = 3.5, df = 3/338, P = 0.02), and future perspectives (stage III < stages I & II, F = 3.5, df = 3/329, P = 0.02). Those who had surgery had significantly less complaints about diarrhea (t = 2.8, df = 268, P = 0.005), but more breast symptoms (t = 2.1, df = 227, P < 0.04).

Those who received radiotherapy had significantly more problems with fatigue (t = 2.2, df = 185, P = 0.03), breast symptoms (t = 2.1, df = 159, P = 0.04), arm symptoms (t = 2.3, df = 175, P = 0.02), and future perspectives (t = 2.3, df = 187, P = 0.02).

There was no significant consistent trend in the association between scale scores and duration of last treatment for surgery and radiotherapy.

In ANCOVA, with age, stage of cancer, marital status, occupation, and duration since last chemotherapy as covariates, age was a significant covariate for sexual functioning (F = 5.7, P < 0.02); marital status was a significant covariate for sexual enjoyment (F = 5.5, P < 0.02); and time since chemotherapy was a significant covariate for future perspectives (F = 6.1, P < 0.02) and body image (F = 4.9, P < 0.03).

### Predictors of global QOL and functional scale scores (Table 4)

**Table 4:** Predictors of functional health status and global quality of life

Dependent variable	Predictors or independent variables	% Variance	Total vari- ance	Standardized Beta	T value	P level
Global QOL & General health	Social functioning	11.6	29.7	-0.31	3.2	0.002
	Sexual enjoyment	4.5		-0.26	3.1	0.003
	Pain	4.4		-0.33	3.5	0.001
	Financial difficulty	4.1		0.24	2.8	0.007
	Age	5.2		-0.14	1.9	0.07
Physical functioning	Occupational status	2.2	53.3	0.9	1.7	0.09
	Role functioning	45.7		0.51	8.0	0.0001
	Cognitive functioning	5.4		0.29	4.6	0.0001
Role functioning	Physical functioning	47.2	67.7	0.31	5.2	0.0001
	Cognitive function	5.5		0.01	0.14	0.89
	Social function	1.8		0.07	1.2	0.22
	Pain	7.2		-0.30	4.5	0.0001
	Fatigue	1.8		-0.11	1.7	0.09
	Dyspnoea	1.9		-0.19	3.3	0.001
	Appetite loss	1.2		-0.15	2.9	0.004
	Constipation	1.0		0.13	2.3	0.02
Emotional functioning	Cognitive functioning	59.6	67.7	0.48	7.4	0.0001
	Social function	3.3		0.18	3.4	0.001
	Pain	1.8		-0.11	1.8	0.08
	Nausea & vomiting	0.08		-0.0.5	0.8	0.42
	Constipation	0.02		-0.19	3.6	0.0001
Cognitive functioning	Emotional functioning	59.6	69.4	0.47	7.6	0.0001
	Physical function	6.7		0.21	3.9	0.0001
	Social function	1.2		0.12	2.2	0.03
	Nausea & vomiting	1.2		-0.11	2.1	0.04
	Pain	0.7		-0.13	2.1	0.04
Social functioning	Marital status	2.2	47.3	-0.11	2.0	0.05
	Emotional functioning	35.1		3.5	5.1	0.0001
	Role function	4.4		0.11	1.5	0.14
	Fatigue	2.3		-0.19	2.8	0.006
	Finance difficulty	3.4		-0.21	3.4	0.001

In stepwise regression analyses, with the GQOL and functional scales, each, as dependent variables, the following trends emerged: First, the socio-demographic variables that entered the regression equations (i.e., age, occupational status and marital status) played rather marginal roles (i.e., variance 2.2 – 5.2%) in predicting GQOL, physical functioning and social functioning, respectively. Second, the clinical variables did not enter the equations. Third, the functional scales were more important (i.e., accounted for the highest proportion of variance) than the symptom scales. Fourth, the variables that accounted for the highest proportion of variance for each scale were as follows: (i) for global QOL, social functioning; (ii) for physical functioning, role functioning; (iii) for role functioning, physical functioning; (iv) for emotional functioning, cognitive functioning; (v) for cognitive functioning, emotional functioning; and (vi) for social functioning, emotional functioning. Fifth, financial difficulty was a highly significant predictor of global QOL (P < 0.007) and social functioning (P < 0.001).

## Discussion

### Profile of EORTC QLQ – C30 and BR-23 scores

We assessed 348 predominantly middle aged Arab women who were mostly receiving chemotherapy. The mean scores for QLQ – C30 and BR-23 indicated that the patients had poor to average functioning and intense symptom experience. We shall now examine the significance of these findings in the context of the international data.

The literature on EORTC QLQ has emphasized that comparison of data should go beyond the usual presentation of mean scores and significant differences [4,35,39,40]. Towards this end, Table 2 shows that, although the mean scores for QLQ – C30 indicated that the patients had poor to average scale scores, only 5.8% to 11.2% had scores that met the ≤ 33% criterion for problematic functioning, while 12.0% to 40.0% met the >66% criterion for more severe symptoms. In other words, despite the reality and popular perception of cancer as a killer disease, many patients undergoing effective modern treatment can expect to have tolerable levels of physical, psychological and social functioning and subjective wellbeing [41-43]. This was particularly the case for the functional scales of the BR-23, where majority of subjects had scores indicating good functioning. It is interesting that, despite their condition, about one-third were hopeful about the future. These findings constitute an evidence base for the country's cancer control program, to boost national health education about prognosis in cancer [8].

With regard to the pattern of functional scale scores of the QLQ – C30, the pattern of responses of our patients was similar to the international data for moderately extensive/severe disease, because the lowest scores were noted for physical functioning and role functioning, while the highest scores were for cognitive and social functioning [44]. This is in line with the reality of a severely debilitating physical illness, where the much available family social support and institutional support can help to boost psychosocial wellbeing [28].

### Factors associated with HRQOL

Of the factors investigated, the noteworthy associations with HRQOL in univariate analyses were age, stage of cancer, receipt of radiotherapy and fatigue. Although the correlations of age with scale scores were rather low, the results are in line with the international data in showing that younger women with cancer tend to have poorer functioning and more intense symptoms, especially if they are on chemotherapy [12-16]. It has been suggested that younger patients are more likely to suffer adverse effects because of induction of early menopause and possible infertility [12]. While the finding of association of disease stage with HRQOL scale scores indicates that the questionnaire has known groups validity, the significant associations were only for three scales (role functioning, diarrhea and future perspective). In the studies that found no significant association between QOL and cancer stage at diagnosis, it has been suggested that the diagnosis of breast cancer is so stressful that it may result in a pattern of psychological morbidity for women in early stages that is similar to that experienced by women with more advanced disease [15]. Coupled with the indication that future perspectives and body image perceptions were better for those who had last treatment over a month ago, [12,45], the above findings should be an evidence base for the contents of a cancer counseling clinic for our patients.

### Predictors of HRQOL

The highlight of our regression analyses is that, contrary to the third hypothesis[30], the functional scale scores were more important in predicting functional scales, than the scores of the symptom scales, while social functioning accounted for the highest proportion of variance in GQOL. This is in line with a Canadian report, which indicated that social functioning was strongly correlated with global QOL, while physical and role functioning were highly correlated [46]. This result has been explained from the perspective of the phenomenon of response -shift [45], which occurs if the patient's perception of the severity of the symptoms changes over time. It is suggested that many patients gradually adapt to the situation following diagnosis and treatment, becoming better at coping with symptoms, such that the patients no longer perceive the burden of the symptoms to be as great as the first time [45]. Hence, for such patients, the physical, emotional and social limitations of the illness tend to become more important determinants of QOL rather than the physical symptoms. However, in a German study that assessed the role of the symptom scales in predicting HRQOL, it was found that fatigue was the most important predictor of QOL [47]. While pain was the only symptom scale that entered our regression model for GQOL, we note that, in univariate analysis, fatigue had the highest correlation with GQOL, of the symptom scales of the QLQ-C30; and fatigue was a significant predictor for social functioning (2.3% variance, P < 0.006). Hence, clinicians should pay particular attention to how to alleviate fatigue, in addition to the usual emphasis on pain [47].

The predictive power of financial difficulty shows that, despite institutional supports, cancer is associated with significant family financial burden. It appears that, although treatment was provided free -of -charge, families still made significant out -of -pocket expenses.

### Comparison with the international data (additional file 1)

The relative youth of our subjects, in comparison with the international data, is noteworthy. The life expectancy at birth for Kuwaiti women is 78.95 years (2009 estimate: https://www.cia.gov/library/publications/the-world-factbook/geos/KU.html). The following pattern emerged in comparing our scale scores with those of the international EORTC QLQ data (additional file 1)[48,49], as well as previous Kuwaiti QOL data from a general population study [50] and women with multiple sclerosis [32], using the global QOL scale of the 26-item WHOQOL-Bref. First, for the functional scales of the QLQ – C30, the Kuwaiti global QOL score was less than those of other countries by at least 10%. In particular, the GQOL score from the neighboring UAE was 20% higher, while that for three Islamic countries was 14% higher. The pattern was the same for physical and role functioning, except role functioning data from China [24]. For emotional functioning, Kuwaiti scores were at least 10% less than the data from Korea, Sweden, and other Western nations. The tendency for the emotional functioning data from the UAE and three Islamic countries to be higher than that from Kuwait, did not reach significance (Effect size: 0.22, 95% C.I. = -0.02 – 0.46). For cognitive and social functioning, Kuwaiti scores were at least 10% less than the data from the UAE, the three Islamic countries and the Western nations.

Second, for the symptom scales of the QLQ – C30, the Kuwaiti data indicated more intense symptoms for dyspnoea, appetite and diarrhea and more financial difficulty (i.e., at least 10% difference).

Third, for the functional scales of the BR – 23, our data was at the middle of the range of the international data for body image, being much similar to the data from Iran and China, indicating better functioning than the Korean data (over 20% difference), but worse functioning than most data from the Western world. For sexual functioning, the Kuwaiti data was at the top of the range of the international data, being similar to the Iranian and UAE data, but indicating better functioning for most data from the Western world. Fourth, of the symptom scales of the BR-23, the mean scores indicated that Kuwaiti women had more intense symptoms than women from all the comparison countries for systemic side effects, breast symptoms and arm symptoms. Regarding systemic side effects, Kuwaiti women had at least 10% difference with women from the Western world, Korea, and Iran, but similar scores with women from the UAE (Effect size, 95% C.I: 0.09, -0.15 -0.33). The pattern was the same for breast symptoms, except that the Kuwaiti women had clinically significantly more intense symptoms than the women from the UAE.

Fifth, using the 0–100% scale of the WHOQOL-Bref[32,50], Kuwaiti women in the general population, women in the general population who rated themselves as being sick, and women attending clinic for multiple sclerosis, all had significantly higher global QOL scores than the women with breast cancer (Effect size, 95% C.I: 1.22, 1.10 – 1.35; and 0.83, 0.60 – 1.05, respectively. Comparison Kuwaiti data were adjusted for socio-demographic variables).

In summary, the profile of scale mean scores for QLQ – C30 indicated that Kuwaiti women had clinically significantly poorer global QOL and functional scale scores, and more intense symptom experience for most scales, in comparison with the international data, including data from neighboring Islamic countries. For the breast specific scale (BR-23), Kuwaiti women seemed to have clinically significantly better sexual functioning, but more severe treatment side effect symptom experience, especially systemic side effects and breast symptoms. The poor global QOL score of Kuwaiti women with cancer was confirmed by the fact that they had significantly lower scores than their compatriots in the general population and those with multiple sclerosis. It appears, therefore, that the experience of clinically more severe symptoms among Kuwaiti women, as a group, was a significant contributor to their low global QOL and comparatively poorer physical, emotional and social functioning [30]. In view of this, clinicians treating Kuwaiti women for cancer should take particular interest in preparing the patients for the acute toxicities of breast cancer treatment [12]. Other factors that could have contributed to their comparatively poor HRQOL are, their relative youth, the fact that they were on chemotherapy [12-16], and the aggressive histopathologic picture of breast cancer in Kuwait [27]. In other words, the biological and treatment side effect factors seemed to be more important than family and institutional supports in the cross-sectional HRQOL outcome of our patients. A longitudinal study is needed to confirm this trend. However, we note that the laudable institutional supports did not include any program on psycho-oncology. Our general experience is that women with breast cancer do not receive any formal psycho-education on the nature of the illness, the implications of treatment and how to cope with cancer. Our findings, therefore, call for a formal program of psycho-oncology at the KCCC, to counsel patients on the nature of the disease and treatment side effects, address their psychosocial concerns and explore methods of coping [51].

### Limitations and strengths of the study

Our findings cannot be generalized to the general population of women with breast cancer because the study was cross-sectional, the participants were a selected clinic population, and there is no general population reference data for the EORTC QLQ – C30 in Kuwait [52]. In addition, we did not sort the patients into categories of disease progression and disease free survivors [28], and the fact that the responses were based on interview could have introduced a bias for patients who may have wished to respond privately. However, we have used an internationally well validated questionnaire to describe the HRQOL of a fairly large number of breast cancer survivors, and we have presented our results in such a way as to make them comparable with data from other countries. Also, there is published data on general population norms for a popular QOL instrument (WHOQOL -BREF) that was presented using the 0 – 100% scale (or the percentage scale maximum method – % SM) [50]. The value of the %SM measure is that it can be used for making comparison with the scale scores of other questionnaires [53].

Of the Arabic samples reported in the literature [9,10], ours is the largest. Furthermore, we categorized scale scores into those with/without significant problems [20,28,37], and applied the principle of clinically significant difference in scale scores between groups [35] to compare our results with the international data. Hence, we met the need to provide clinically-based benchmarks in interpreting QOL data, so as to make them meaningful to clinicians[4,5,44]. Finally, it appears that our patients had similar demographic characteristics with those of breast cancer clinic populations in Kuwait [27,38].

## Conclusion

The relatively high number that met the criterion for good functioning on the physical, role, emotional, cognitive, social, and sexual functioning scales is an evidence base to boost national health education about psychosocial prognosis in cancer. Their sense of optimism in the face of such a devastating disease is particularly noteworthy. However, in view of their poor performance on the symptom scales, clinicians treating Kuwaiti women with breast cancer should take particular interest in preparing the patients for the acute toxicities of treatment, as well as addressing fatigue and pain [12,47]. The findings call for the institution of a psycho-oncology service at the KCCC, to address psycho-social outcome.

## Competing interests

The authors declare that they have no competing interests.

## Authors' contributions

SA conceived the study. SA and JUO jointly designed the study, analyzed the data and wrote up the manuscript. SA supervised data collection. Both authors read and approved the manuscript.

## Pre-publication history

The pre-publication history for this paper can be accessed here:

http://www.biomedcentral.com/1471-2407/9/222/prepub

## Supplementary Material

Additional file 1 **Tables five – seven in landscape**. The data provided compared the EORTC QLQ – C30 and BR – 23 scores from several countries with our results. Click here for file
