# Supplementary material for: Health – related quality of life of Kuwaiti women with breast cancer: a comparative study using the EORTC Quality of Life Questionnaire
Source: BMC Cancer. 2009 Jul 8;9:222. doi: 10.1186/1471-2407-9-222 (PMC2714557; doi:10.1186/1471-2407-9-222)
Supplement: Additional file 1 — Tables five – seven in landscape. The data provided compared the EORTC QLQ – C30 and BR – 23 scores from several countries with our results. [file 1471-2407-9-222-S1.doc]

Additional file for Tables in landscape:

Table 5: International comparisons: QLQ –C30: Mean scores of functional scales from other countries in comparison with Kuwait

| Country/ (N) | Authors | Mean age | Global QOL | Physical  functioning | Role func  -tioning | Emotional  functioning | Cognitive  functioning | Social fun-  ctioning |
| --- | --- | --- | --- | --- | --- | --- | --- | --- |
| United Arab  Emirate (N = 87) | Awad et al  [9] | 48.6 (9.9) | 74.6(18.0) | 73.8(22.6) | 82.5(27.6) | 65.8(32.8) | 74.0(30.7) | 85.1(26.0) |
| S. Korea (N =593; chemother | Ahn et al.,  [20] | 46.6 &  47.8 | 66.5 | 77.2 | 74.3 | 71.8 | 71.2 | 77.5 |
| China(N =233) | Wan et al [24] | 48.6 | 52.3 | 69.8 | 50.16 | 60.2 | 62.18 | 51.76 |
| Germany(N= 1927) | Waldmann et  al., [28] | 58.8 | 65.5 | 93.2 | 62.1 | 62.2 | 78.0 | 71.4 |
| Sweden: general  Population(N=3069): cancer pts | Michelson et  al., [52] | - | 73.2 | 81.5 | 81.6 | 81.5 | 86.6 | 87.5 |
| USA (N = 1357) | Janz et al [15] | 60.0 | - | 75.0 | 72.5 | 70.0 | - | 77.0 |
| UK (N = 2180) | Hopwood et  al.,[12] | 56.9 | 66.8 &  69.8 | 83.8 | 70.1 | 75.3 | 81.9 | 75.6 |
| Italy (N = 604) | Apolone et al  [53] | 62.6 | 90.7 | 78.4 | 84.0 | 78.1 | 81.6 | 90.7 |
| 34 countries vs  UK (N = 3069) | Scott et al  [10] | 51.6 -63.0 | 55.0 -69.2 | 74.6 -86.0 | 64.3 -82.2 | 59.7 -75.0 | 76.9 -87.5 | 70.3 -83.8 |
| 3 Islamic states:  Turkey, Iran, Egypt vs UK (N= 2800) | Scott et al [10] | 51.6 (13.7) | 59.9 | 78.5 | 71.2 | 65.7(25.0) | 81.4 | 76.9 |
| Norway: all cancers in general popn  (N = 91) | Hjermstad  et al.[38] | 65.9 | 59.7 | 72.0 | 77.6 & 61.7 | 77.9 | 76.8 | 70.4 |
| Kuwait(N =348) | Present study | 48.3 | 45.0 | 52.7 | 55.1 | 60.2 | 59.4 | 61.3 |

Table 6: International comparisons: QLQ – C30, mean scores of symptom scales for other countries compared with Kuwait*

| Country/ (N) | Authors | Fatigue | Nausea  & vomiting | Pain | Dyspnoea | Sleep | Appetite | Consti  pation | Diarrhea | Financial  difficulty |
| --- | --- | --- | --- | --- | --- | --- | --- | --- | --- | --- |
| United Arab  Emirate (N = 87) | Awad et al  [9] | 35.8(21.8) | 21.8(33.4) | 85.0(  26.9) | 21.1(30.1) | 44.7(  42.7) | 31.8(39.3) | 25.2  (37.9) | 11.2(27.8) | 10.5(  26.7) |
| S. Korea (N =593) | Ahn et al [20] | 37.6 | 9.6 | 20.0 | 18.6 | 27.4 | 10.3 | 23.1 | 8.7 | 25.2 |
| China(N =233) | Wan et al [24] | 47.1 | 38.6 | 42.8 | 25.1 | 42.2 | 44.6 | 30.0 | 18.6 | 61.5 |
| Germany(N= 1927) | Waldmann et  al., [28] | 42.2 | 6.8 | 29.1 | 28.0 | 45.5 | 11.7 | 13.6 | 7.1 | 21.7 |
| Sweden: general  population cancer pts | Michelson et  al.,[52] | 27.0 | - | 25.7 | 25.4 | 24.9 | - | - | - | 7.6 |
| USA (N = 1357) | Janz et al[15] | 58 | - | - | - | - | - | - | - | - |
| UK (N = 2180) | Hopwood et  al., [12] | 33.1 | 7.1 | 20.9 | 13.4 | 33.8 | 12.1 | 14.7 | 6.3 | 15.4 |
| Italy (N = 604) | Apolone et al  [53] | 19.8 | 2.6 | 17.2 | - | - | - | - | - | - |
| Norway: all cancers in general popn  (N = 91) | Hjermstad  et al.,[38] | 42.6 | 10.4 | 33.9 | 29.8 | 36.4 | 12.7 | 20.0 | 13.7 | 14.7 |
| Kuwait (N=348)** | Present study | 38.9 | 30.2 | 43.8 | 42.1 | 42.7 | 37.4 | 27.8 | 21.9 | 31.2 |

- *Higher scores indicate more intense symptoms or problems
- ** For each scale, compared with other countries, a clinically important difference is ≥10%

Table 7: International comparisons: QLQ – BR-23, mean scores of functional and symptom scales compared with Kuwait*

| Country/ (N) | Authors | Mean age | Body** image | Sexual** fun  -ctioning | Sexual** enjoy  -ment | Future*** pers-  pective | Systemic side  Effects*** | Breast symptoms  *** | Arm symptoms  *** | Upset by hair loss  *** |
| --- | --- | --- | --- | --- | --- | --- | --- | --- | --- | --- |
| United Arab  Emirate (N = 87) | Awad et al  [9] | 48.6 | 69.4(26.5) | 62.6(28.7) | 59(31.8) | 47.3(39.4) | 38.4(22.6) | 21.4(18.5) | 32.0(31.1) | 63.2(43.5) |
| S. Korea (N =593) | Ahn et al [20] | 46.6  47.8 | 47.2 | 23.2 | 40.2 | 42.5 | 27.8 | 19.1 | 29.1 | 41.3 |
| China(N =233) | Wan et al [24] | 48.6 | 60.9 | 21.0 | 27.8 | 51.3 | 34.1 | 25.9 | 33.1 | 42.7 |
| Germany(N= 1927) | Waldmann et. al.[28] | 58.8 | 73.7 | 29.8 | 69.2 | 45.8 | 28.0 | 24.1 | 32.9 | 59.3 |
| Iran (N= 168 & 151: follow-ups) | Montazeri et al., [19] | 47.2(13.5) | 61.0(33.1) | 75.3(23.8) | 39.8(29.1) | 35.1(26.9) | 30.6(17.4) | 11.1(14.8) | 19.7(17.4) | 67.7(31.4) |
| USA (N = 1357) | Janz et al [15] | 60.0 | 70.0 | 17.0 | 51.0 | 47.0 | - | - | - | - |
| UK (N = 2180) | Hopwood et al [12] | 56.9 | 78.1 | 19.2 | 59.1 | 54.8 | 20.9 | 18.5 | 21.2 | 50.6 |
| Netherlands  (N = 170).  Follow up | Sprangers et al  [18] | 51 (median) | 85.4 | 22.9 | 59.1 | 61.8 | 20.3 | 28.4 | 23.1 | 42.5 |
| Spain  (N = 168) | [18] | 55: median | 92.3 | 9.4 | 50.7 | 65.0 | 9.6 | 18.4 | 14.7 | 34.4 |
| USA (N=158) | [18] | 52: median | 63.0 | 23.9 | 47.1 | 45.1 | 22.2 | 21.7 | 30.4 | 46.2 |
| Kuwait(N=348) | Present |  | 61.8 | 70.2 | 61.8 | 59.7 | 40.2 | 35.3 | 38.2 | 44.4 |

*For each scale, compared with other countries, a clinically important difference is ≥10%

** Higher scores indicate better functioning. *** Higher scores indicate more intense symptoms or problems.
